# Supplementary material for: Stokes flow analogous to viscous electron current in graphene
Source: Nat Commun. 2019 Feb 26;10:937. doi: 10.1038/s41467-019-08916-5 (PMC6391415; doi:10.1038/s41467-019-08916-5)
Supplement: Supplementary file 1 — Supplementary Information [file 41467_2019_8916_MOESM1_ESM.pdf]

# Stokes flow analogous to viscous electron current in graphene

Mayzel et al.

**Supplementary Note 1: Disk Geometry Analytic Solution**

The constitutive equation governing the steady viscous flow of an incompressible Newtonian fluid is the Stoke's law: $\eta\nabla^2\mathbf{u} = \nabla P$. Since the system is two-dimensional we introduce streamline function $\psi(x, y)$ via $\mathbf{u} = \mathbf{z}\times\nabla\psi$ that reduces the Stokes equation to a bi-hamonic equation $(\nabla^2)^2\psi = 0$. A solution for the bi-harmonic equation in polar coordinates $(r, \theta)$ is given by:

$$\psi(r,\theta) = \frac{\left(r^2-R^2\right)^2}{2\pi R}\left[\int_0^{2\pi}\frac{(R-r\cos(\theta'-\theta))f(\theta')}{\left(R^2+r^2-2Rr\cos(\theta'-\theta)\right)^2}d\theta' - \frac{1}{2}\int_0^{2\pi}\frac{g(\theta')}{R^2+r^2-2Rr\cos(\theta'-\theta)}d\theta'\right],$$

where $R$ is the disk radius and on the boundaries $\psi$ satisfies: $\psi(r = R, \theta) = f(\theta)$ and $\partial_r\psi\big|_{r=R} = g(\theta)$. For no-slip boundary conditions with flow injected and collected with velocity $u_0$ at $(0, \pm R)$, $g(\theta) = 0$ and $f(\theta) = Ru_0\left(\Theta\left(\theta-\pi/2\right) - \Theta\left(\theta-3\pi/2\right)\right)$, where $\Theta$ is the Heaviside step function. Thus, the resulting streamline function is given by:

$$\psi(r,\theta) = \frac{u_0R}{\pi}\left[\tan^{-1}\left(\frac{(r+R)\cot\left(\frac{2\theta+\pi}{4}\right)}{(r-R)}\right) + \tan^{-1}\left(\frac{(r+R)\tan\left(\frac{2\theta+\pi}{4}\right)}{(r-R)}\right)\right] + \frac{u_0 r}{\pi}\left[\frac{(r^4-R^4)\cos\theta}{r^4+R^4+2r^2R^2\cos 2\theta}\right].$$

Solving for the pressure field $P(x, y)$ from $\psi$ we find:

$$P(x,y) = \eta u_0\Re\left(\frac{8iR^2}{\pi}\frac{\bar{z}}{(\bar{z}^2+R^2)^2}\right),$$

where $\bar{z} = x - iy$.

Supplementary Figure 1a shows the velocity magnitude of the flow in the disk geometry ($e = 0$) and vortices are absent, which is the case for Ohmic flow. Correspondingly the velocity profile for different $y/w$ is shown in Supplementary Figure 1b. However, the pressure field $P(x, y)$ shows an interesting pattern: along the $y/w = 0$ line the pressure is constant, i.e. $P(x, y = 0) = P_0$, and along constant $y/w$ values in the upper half disk the pressure on the edges is lower than $P_0$ and in the center it is higher than $P_0$ (Supplementary Figure 1c). This trend is opposite in the lower half disk, which creates a pressure gradient −unique to viscous flow− resulting in a force that counteracts the viscous shear forces in the flow.

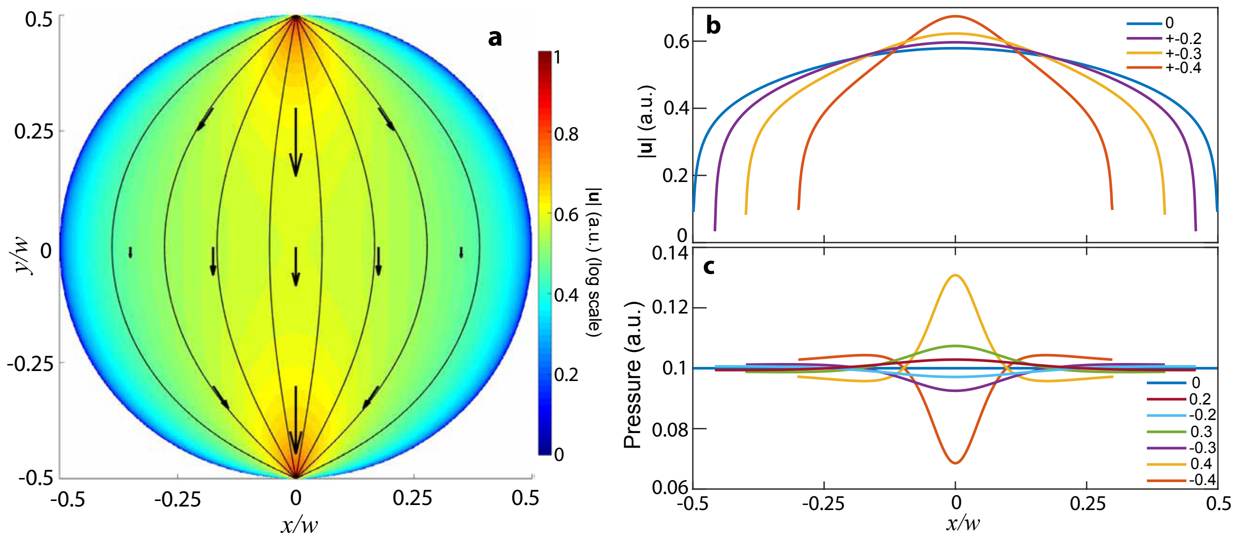


**Supplementary Figure 1:** (**a**) Color map of fluid velocity magnitude $|\mathbf{u}|$ on log scale for a disk $e = 0$. Streamlines and flow direction from inlet to outlet are shown by lines and arrows, respectively. Spatial variation, for a disk, of (**b**) velocity magnitude $|\mathbf{u}|$ and (**c**) pressure $P$, as a function of $x/w$ for different $y/w$ values (shown by different colored lines).

## Supplementary Note 2: Elliptic Geometry Computational Method

To obtain the streamline function $\psi(x, y)$ without solving the bi-harmonic equation directly, we define the vorticity of the flow field as $\omega = \nabla \times \mathbf{u}$, which is used to obtain a complex harmonic function $\Phi = \frac{1}{\eta}P + i\omega$. Since $\frac{1}{\eta}P$ and $\omega$ form a Cauchy-Riemann pair, we can use conformal mapping to map $\Phi$ from a disk to an ellipse. To obtain the streamline function $\Phi$ is not enough, since the boundary conditions of the problem are imposed on $\psi$. Therefore, a multi-step computational approach, involving conformal mapping on $\Phi$, was implemented, which is outlined below:

1. **Conformal mapping** - By using a conformal mapping on the complex harmonic function $\Phi$ from a disk to an ellipse, we get the pressure field and the vorticity of an ellipse.

2. **Inverse Laplacian** - Numerically inverting the relation $\omega = \nabla^2\psi$ in order to get $\psi$ up to a harmonic function.

3. **General form of a harmonic function** - Solving Laplace equation in elliptic coordi-

nates to get the most general form of a harmonic function that satisfies the symmetry considerations in the problem.

4. **Fitting harmonic function using boundary conditions** - Fitting the coefficients of the general form of the harmonic function using the no-slip boundary conditions.

Then by adding the fitted harmonic function and the inverted Laplacian, we obtain the streamline function $\psi$. In the next section, we elaborate upon each step of this approach.

### A. Ellipse definition and the Elliptic coordinate system

An ellipse in cartesian coordinates $(x, y)$ is defined as

$$\frac{x^2}{a^2} + \frac{y^2}{b^2} = 1,$$

where $a$ and $b$ are the major and minor axes of the ellipse, respectively. The eccentricity of the ellipse is defined by: $e = \sqrt{1 - \frac{b^2}{a^2}}$. When $e = 0$ we get a disk, when $e \to 1$ we get an infinite line or strip. When solving algebra in the elliptic geometry we use the transformation of cartesian coordinates $(x, y)$ to elliptic coordinates $(\mu, \nu)$:

$$x = c \cosh \mu \cos \nu; \qquad y = c \sinh \mu \sin \nu,$$

where $\mu$ is a nonnegative real number, $\nu \in [0, 2\pi]$, and $c$ is a scaling factor. In our case, we deal with a problem inside the ellipse where we fix $b = 1$ and vary the major axis $a$, such that the scaling factor is given by $c = \sqrt{a^2 - 1}$.

### B. Conformal mapping

Since the pressure field $P$ and the vorticity $\omega$ form a Cauchy-Riemann pair, we can define a complex function $\Phi(\bar{z}) = \frac{1}{\eta} P + i\omega$ and use conformal mapping to get $\Phi$ of the ellipse from $\Phi$ of the disk. The conformal mapping formula is given by:

$$\kappa = (m)^{1/4} \mathrm{sn}\left( \frac{2K(m)}{\pi} \sin^{-1} \left( \frac{z}{\sqrt{a^2 - 1}} \right), m \right),$$

where sn is the Jacobian elliptic sine, and $K(m)$ is the complete elliptic integral of the first kind with modulus $m$. This formula maps the interior of the ellipse in the $z$-complex plane

to the interior of a disk in the $\kappa$-complex plane. The relation between $m$ and the ellipse major axis $a$ is given by:

$$\frac{K(m')}{K(m)} = \frac{2}{\pi}\sinh^{-1}\left(\frac{2a}{a^2-1}\right); \quad a = \coth\left(\frac{\pi}{4}\frac{K(m')}{K(m)}\right),$$

where $m' = 1 - m$, and the ellipse minor axis is set to $b = 1$.

### C. Inverse Laplacian

Then the real part of the vorticity $\omega$ is extracted from $\Phi$ as $\omega = \Re(\Phi)$, and an inverse Laplacian is applied numerically on $\omega$. This was done by performing a Fast Fourier Transform (FFT) on $\omega$: $\tilde{\omega}(\mathbf{k}) = FFT[\omega(x,y)]$. Dividing $\tilde{\omega}(\mathbf{k})$ by $-k^2$, and applying an inverse FFT back to obtain $\psi$ up to a harmonic function, $\psi_0 = FFT^{-1}[-\tilde{\omega}(\mathbf{k})/k^2]$.

### D. Harmonic function solution

Since $\omega = \nabla^2\psi$, applying the inverse Laplacian on $\omega$ results in $\psi$ up to a harmonic function, $\psi = \psi_0 + F$. Here $\psi_0 = (\nabla^2)^{-1}\omega$ is the result of the inverse Laplacian and $F$ is a general harmonic function in elliptic coordinates, i.e. $\nabla^2 F = 0$. The general harmonic function that upholds the symmetry considerations of the problem is given by:

$$F(\mu,\nu) = \sum_{n=0}^{N}\left(A_n e^{-n\mu} + B_n e^{n\mu}\right)\cos n\nu.$$

$A_n$ and $B_n$ are coefficients that would be determined by fitting $\psi_0 + F$ to the boundary conditions of the problem.

### E. Fitting harmonic function using boundary conditions

The values of $A_n$ and $B_n$ for each order $N$ are then determined by fitting $\psi_0 + F$ to the boundary conditions. This is done in MATLAB using fminsearch function, up to a predetermined order $N$, by minimizing the difference between the analytic boundary conditions to the boundary conditions values of $\psi_0 + F$ resulting from the choice of $A_n$ and $B_n$.

## Supplementary Note 3: Additional results for the Elliptic Geometry

Supplementary Figures 2b and 3b shows additional results from numerics for the elliptic geometry for $e = 0.9$ and $e = 0.95$, corresponding to the experimental results shown in Supplementary Figures 2a and 3a, respectively.

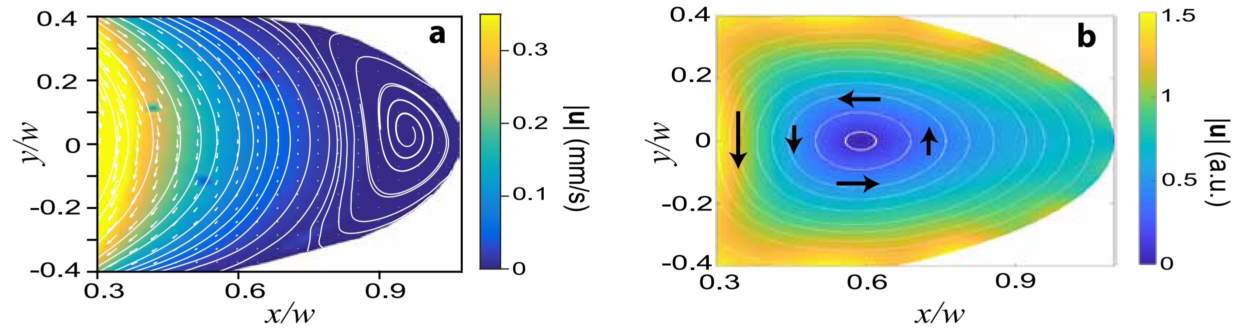


**Supplementary Figure 2**: Color map of fluid velocity magnitude $|\mathbf{u}|$ for elliptic cavity of $e = 0.9$ from (**a**) experiment and (**b**) numeric for $N = 20$. Streamlines and flow direction are shown by lines and arrows, respectively.

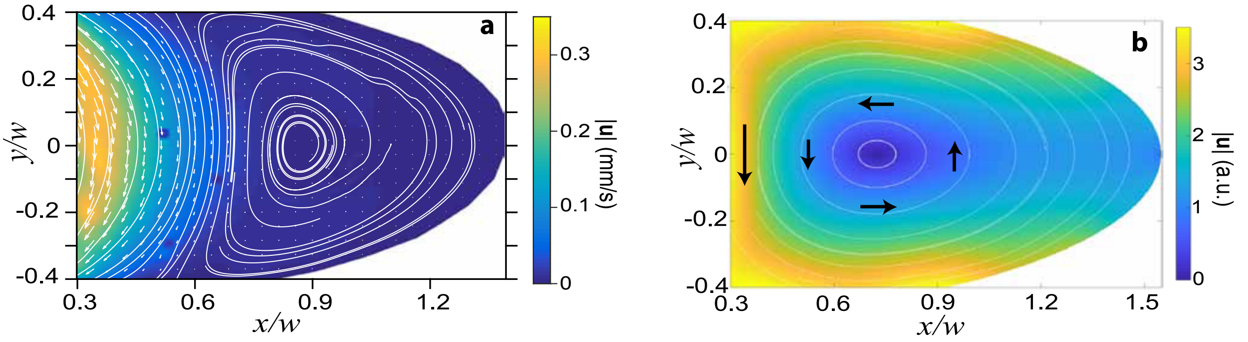


**Supplementary Figure 3**: Color map of fluid velocity magnitude $|\mathbf{u}|$ for elliptic cavity of $e = 0.95$ from (**a**) experiment and (**b**) numeric for $N = 16$. Streamlines and flow direction are shown by lines and arrows, respectively.

Supplementary Figure 4 compares the divergence fields computed from experimentally and numerically obtained velocity fields for $e = 0.97$. Supplementary Figure 5 shows the divergence field obtained from the experimental results for $e = 0.9$ and 0.95. It is close to zero in the cavity regions which suggests an effective two-dimensional flow and the non-zero

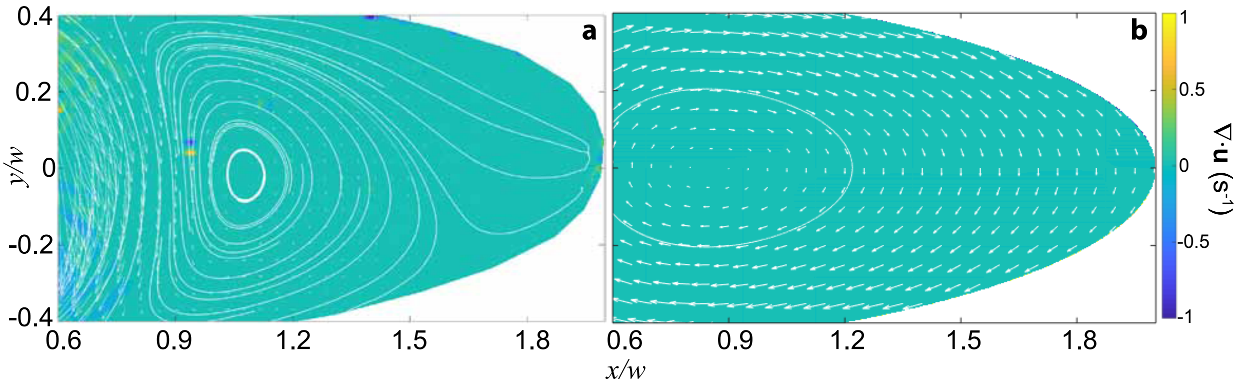


**Supplementary Figure** 4: Divergence field (in color) computed from (**a**) experimentally and (**b**) numerically obtained velocity field (shown by arrows) for $e = 0.97$. Streamlines are shown by white lines.

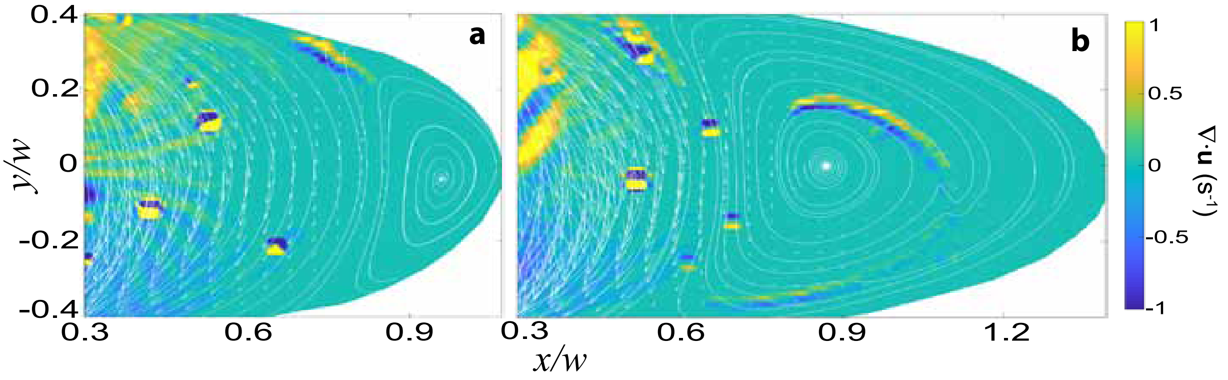


**Supplementary Figure** 5: Divergence field (in color) obtained from experimentally measured velocity field (shown by arrows) for cavities of (**a**) $e = 0.9$ and (**b**) $e = 0.95$. Streamlines are shown by white lines.

deviations appear in plus/minus pairs, suggesting velocity fluctuations, or lower accuracy of PIV measurements in the regions where the velocities are high.
