# Supplementary material for: Stokes flow analogous to viscous electron current in graphene
Source: Nat Commun. 2019 Feb 26;10:937. doi: 10.1038/s41467-019-08916-5 (PMC6391415; doi:10.1038/s41467-019-08916-5)
Supplement: Supplementary file 3 — Description of Additional Supplementary Files [file 41467_2019_8916_MOESM3_ESM.pdf]

Supplementary Movie 1:
The movie shows the vortex flow in a rectangular cavity ($e \rightarrow 1$) at Re=0.07. An exposure time of 15 s is used to record the movie. The movie is displayed at 10 fps. Two arrows indicate the flow direction.

Supplementary Movie 2:
The movie shows the flow in an elliptical cavity (e=0.95) at Re=0.08. An exposure time of 15 s is used to record the movie. The movie is displayed at 10 fps. Two arrows indicate the flow direction.

Supplementary Movie 3:
The movie shows the flow in an elliptical cavity (e=0.9) at Re=0.07. An exposure time of 15 s is used to record the movie. The movie is displayed at 10 fps. Two arrows indicate the flow direction.

Supplementary Movie 4:
The movie shows the flow in an elliptical cavity (e=0.75) at Re=0.02. An exposure time of 5 s is used to record the movie. The movie is displayed at 10 fps. Two arrows indicate the flow direction.
